# Comment on: Protocolized care pathways in emergency general surgery: a systematic review and meta-analysis

**DOI:** 10.1093/bjs/znae234

**Published:** 2024-09-18

**Authors:** Hua Luo, Yongwei Su, Yu Ren

**Affiliations:** Department of Orthopaedics, Taizhou Hospital of Zhejiang Province affiliated to Wenzhou Medical University, Taizhou, China; Department of Orthopaedics, First Affiliated Hospital of Jinzhou Medical University, Jinzhou, China; Department of Pharmacy, Taizhou Hospital of Zhejiang Province affiliated to Wenzhou Medical University, Taizhou, China

*Dear Editor*

We read with interest the article by Harji *et al*.^1^. The study suggests that protocolized care pathways reduce hospital stays, complications such as postoperative pneumonia and infections, and improve gastrointestinal function. We appreciate the authors’ efforts but noticed several issues that need addressing.

First, the authors mentioned that ‘Four RCTs reported mean length of hospital… Protocolized care pathways were associated with a significantly shorter length of stay…… (SMD −2.47……*P* = 0.002; *Fig. 2*) in result’. However, *Fig. 2* includes five studies (4 RCTs and 1 retrospective study). This appears to be an error, and the summary figure should include only the RCTs, as shown in our corrected *Fig. S1*.

Second, several outcomes show high heterogeneity (length of stay *I*² = 97%, time to flatus *I*² = 96%, time to defaecation *I*² = 98%, time to diet *I*² = 98%). The authors did not explore the sources of this heterogeneity or conduct sensitivity analyses, which may reduce the reliability of the results.

Third, there are multiple errors in *Fig. 1*. After removing 571 duplicates from 2756 articles, 2185 should remain, but *Fig. 1* shows 2188. Then, 907 articles were excluded, leaving 21 for full-text review, but it is unclear what happened to the remaining 1260 articles.

Fourth, the conclusions do not adequately consider the heterogeneity of the included populations and protocolized care pathways. Significant differences in age, sex, ASA grades, types of surgery, and complexity can influence results. Additionally, variations in pathway components and adherence rates affect comparability and overall analysis. These issues limit the validity and generalizability of the conclusions, as the study does not fully address these heterogeneities.

We acknowledge the authors’ contributions and hope our suggestions help refine their future research. We look forward to their feedback.

## Supplementary Material

znae234_Supplementary_Data
