# Supplementary figures and images for: Comment on: Protocolized care pathways in emergency general surgery: a systematic review and meta-analysis
Source: Br J Surg. 2024 Sep 18;111(9):znae234. doi: 10.1093/bjs/znae234 (PMC11408925; doi:10.1093/bjs/znae234)

**Supplement**


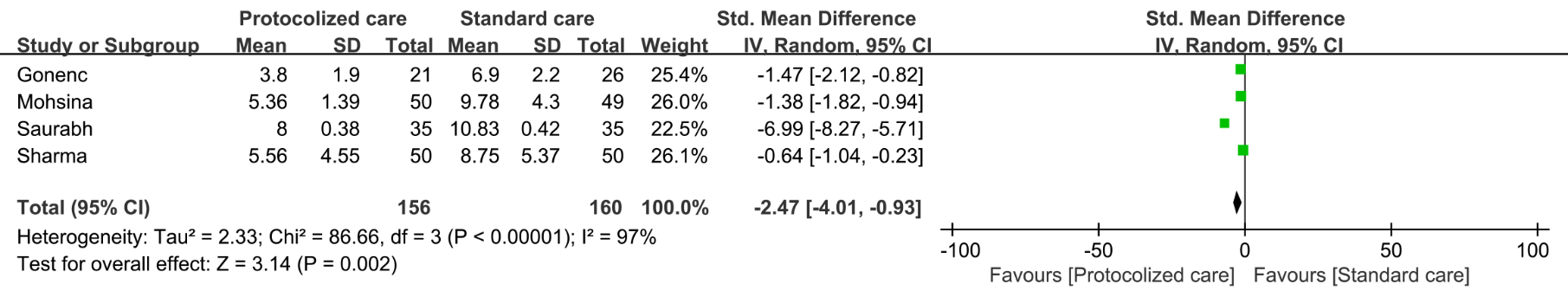
**Figure 1.** Pooled meta-analysis of RCTs forest plot length of stay.

Supplement: znae234_Supplementary_Data [file znae234_supplementary_data.docx]
